# Impact of Residing in Below Median Household Income Districts on Outcomes in Patients with Advanced Barrett’s Esophagus

**DOI:** 10.1093/jcag/gwad018

**Published:** 2023-04-27

**Authors:** Suqing Li, Yusuke Fujiyoshi, Sechiv Jugnundan, Gary May, Norman Marcon, Jeffrey Mosko, Christopher Teshima

**Affiliations:** Division of Gastroenterology and Hepatology, Department of Medicine, University of Calgary, Calgary, Alberta, Canada; Division of Gastroenterology and Hepatology, Department of Medicine, The Center for Advanced Therapeutic Endoscopy and Endoscopic Oncology, St. Michael’s Hospital, Toronto, Ontario, Canada; Division of Gastroenterology and Hepatology, Department of Medicine, The Center for Advanced Therapeutic Endoscopy and Endoscopic Oncology, St. Michael’s Hospital, Toronto, Ontario, Canada; Division of Gastroenterology and Hepatology, Department of Medicine, The Center for Advanced Therapeutic Endoscopy and Endoscopic Oncology, St. Michael’s Hospital, Toronto, Ontario, Canada; Division of Gastroenterology and Hepatology, Department of Medicine, The Center for Advanced Therapeutic Endoscopy and Endoscopic Oncology, St. Michael’s Hospital, Toronto, Ontario, Canada; Division of Gastroenterology and Hepatology, Department of Medicine, The Center for Advanced Therapeutic Endoscopy and Endoscopic Oncology, St. Michael’s Hospital, Toronto, Ontario, Canada; Division of Gastroenterology and Hepatology, Department of Medicine, The Center for Advanced Therapeutic Endoscopy and Endoscopic Oncology, St. Michael’s Hospital, Toronto, Ontario, Canada

**Keywords:** Barrett’s esophagus, Equality, Income, Socioeconomic

## Abstract

**Background:**

Barrett’s esophagus (BE) is a premalignant condition to esophageal adenocarcinoma (EAC). Low socioeconomic (SES) status adversely impacts care and outcomes in patients with EAC, but this has not been evaluated in BE. As the treatment of BE is similarly intensive, we aimed to evaluate the effect of SES on achieving complete eradication of intestinal metaplasia (CE-IM), dysplasia (CE-D) and development of invasive EAC.

**Methods:**

Our study was a retrospective cohort study. Consecutive patients between January 1, 2010, to December 31, 2018, referred for BE-associated high-grade dysplasia or intramucosal adenocarcinoma were included. Pre, intra and post-procedural data were collected. Household income data was collected from the 2016 census based on postal code region. Patients were divided into income groups relative to the 2016 median household income in Ontario. Multivariate regression was performed for outcomes of interest.

**Results:**

Four hundred and fifty-nine patients were included. Rate of CE-IM was similar between income groups. Fifty-five per cent (*n* = 144/264) versus 65% (*n* = 48/264) in the below and above-income groups achieved CE-D, respectively, *P* = 0.02. Eighteen per cent (*n* = 48/264) versus 11% (*n* = 22/195) were found to have invasive EAC during their treatment course in below and above-income groups, respectively, *P* = 0.04. Residing in a below-median-income district was associated with developing invasive EAC (Odds Ratio, [OR] 1.84, 95% confidence interval [CI] 1.01 to 3.35) and failure to achieve CE-D (OR 0.64, 95% CI 0.42 to 0.97).

**Conclusions:**

Residing in low-income districts is associated with worse outcomes in patients with advanced BE. Further research is needed to guide future initiatives to address the potential impact of SES barriers in the optimal care of BE.

## BACKGROUND

Barrett’s esophagus (BE) has been increasing in incidence and is the only known premalignant lesion in the development of esophageal adenocarcinoma (EAC) (1,2). Although prior studies have investigated the impact of socioeconomic status (SES) in cancer care, there remains minimal literature on the effects of SES on outcomes in patients with premalignant conditions such as BE (3). Advanced BE, encompassing patients with BE and high-grade dysplasia (HGD) or intramucosal cancer (IMC), has the highest risk of developing invasive EAC (4). Thus, timely evaluation and initiation of endoscopic eradication therapies (EET) are crucial for all patients with advanced BE to prevent the development of invasive EAC (4).

However, in many areas, consistent access to the specialized care necessary to detect and manage advanced BE may be limited. Poor adherence to quality indicators in BE surveillance and eradication therapy has previously been shown (5). As such, it is suggested that patients with dysplastic BE requiring EET be referred to high-volume centers of expertise (4). However, geographical remoteness, and thus ease of access to expert care centers, has also been linked to lower SES (6). Additionally, prior studies have shown a greater risk of nonadherence to surveillance endoscopy in lower SES patients with non-dysplastic BE (7).

In addition, given the resource-intensive surveillance, treatment, and follow-up regimens necessary to effectively manage advanced BE, a patient’s background SES may result in inequities in access to care and impact outcomes. Notably, increasing risk of EAC and disparities in access to optimal surgical care were associated with low SES in prior studies (8,9). In line with these findings, studies have shown disparities in 5-year survival rates for patients in low SES populations with EAC (10).

Despite these concerns, no prior studies have directly assessed the impact of residing in low SES districts on clinical outcomes in BE. Thus, our study aimed to evaluate the potential effects of SES on outcomes in patients with advanced BE referred to a major tertiary expert referral center. Our primary outcome measures were achievement of complete eradication of intestinal metaplasia (CE-IM), dysplasia (CE-D), and development of invasive EAC. Our institution, located in Toronto, Ontario, is the largest referral center for advanced BE management in Canada, servicing the highest population centers in the country.

## METHODS

### Study Design and Data Extraction

All consecutive patients referred for endoscopic treatment of BE-related HGD/IMC between January 1, 2010 and December 31, 2018, were recruited into a prospectively maintained registry at a single tertiary referral center. Our study was a retrospective cohort study utilizing data accessed via this prospective BE registry. Written informed consent was obtained from each patient for all procedures and participating in the prospective institutional registry. The study was carried out in accordance with the Declaration of Helsinki and was approved by the institutional research ethics committee.

Patients were included in the study if their initial referral histology had confirmed HGD/IMC based on a biopsy or an endoscopic mucosal resection (EMR) specimen agreed upon by at least two gastrointestinal pathologists. Patients were excluded if their primary location of residence was outside the province of Ontario or if they declined or could not provide informed consent to participate.

Baseline demographic characteristics and features of BE at initial endoscopy were recorded. Measurement of the circumferential and maximal length of BE was done based on Prague criteria (11). Lesion and case details were obtained by reviewing the patient’s local electronic health record (EHR). When details were unavailable through the EHR, a physical chart review of the case, including a post hoc review of endoscopic images, was conducted. Patient outcomes were collected up to 2 years from the date of index endoscopy.

All postal codes were collected for each patient. Forward sortation area (FSA) of postal codes were used to obtain values for total median household after-tax income of patients census district based on data from the Canadian 2016 Census profile (12). Patient postal codes were geomapped with ArcGIS Online to provide a descriptive geographical overlay of referral distributions and patient driving/euclidian distances to our institution. Patients were divided into above and below-median provincial income household districts based on the reported median household after-tax income in the province of Ontario from the 2016 census of $65,285 (13). Division of household income levels into quartiles was also performed for purposes of sensitivity analysis (Supplementary Tables 1–3).

### Endoscopy Procedures

All patients in the registry underwent endoscopy by an advanced endoscopist with specialized training in the management of BE, with the goal of performing EET to achieve CE-D and CE-IM. Three basic treatment approaches were utilized based on expert endoscopists’ discretion: either a multimodal approach consisting of targeted EMR/Endoscopic submucosal dissection (ESD) for endoscopically visible mucosal abnormalities followed by RFA, a radical EMR/ESD approach aiming for complete eradication of BE that was continued until no further visible columnar mucosa could be identified, or RFA without EMR/ESD in patients with HGD but no visible discrete lesions.

Patients were recommended to have follow-up endoscopy with repeat EET until achievement of CE-IM. Systematic biopsies were taken of any remaining BE as per Seattle protocol, plus target biopsies were taken from any visible abnormalities (4). A distal attachment cap was utilized to optimize mucosal examination.

All patients were placed on once- or twice-daily proton pump inhibitor therapy. After achieving CE-IM, patients were scheduled for continued endoscopic surveillance at intervals of every 3 to 6 months for one year and then annually thereafter.

### Statistical Analysis

Descriptive categorical variables were summarized as frequencies (%). Continuous variables were expressed as means with standard deviation (SD) or medians with interquartile range (IQR) as appropriate based on distribution. Mann–Witney *U* test and Student’s *t*-test were used to compare medians and means between groups as appropriate. Chi-square was used to compare frequencies between groups. Univariate and multivariate regression were used to analyze potential variables associated with outcomes of interest. Variables selected to be included in the multivariate model were chosen a priori based on our clinical expertise and previously published data (14). All variables included in the multivariate analysis were evaluated via the variance inflation factor (cut-off threshold of 2.5) and correlation coefficients (cut-off threshold of 0.8) to ensure no significant multicollinearity. Missing data were omitted, there was a <15% rate of missing data. All statistical tests were two-sided, with a *P*-value <0.05 considered statistically significant. All analyses were conducted using SPSS version 28.0 (IBM Corporation).

## RESULTS

### Patient Demographics

**Figure 1.**
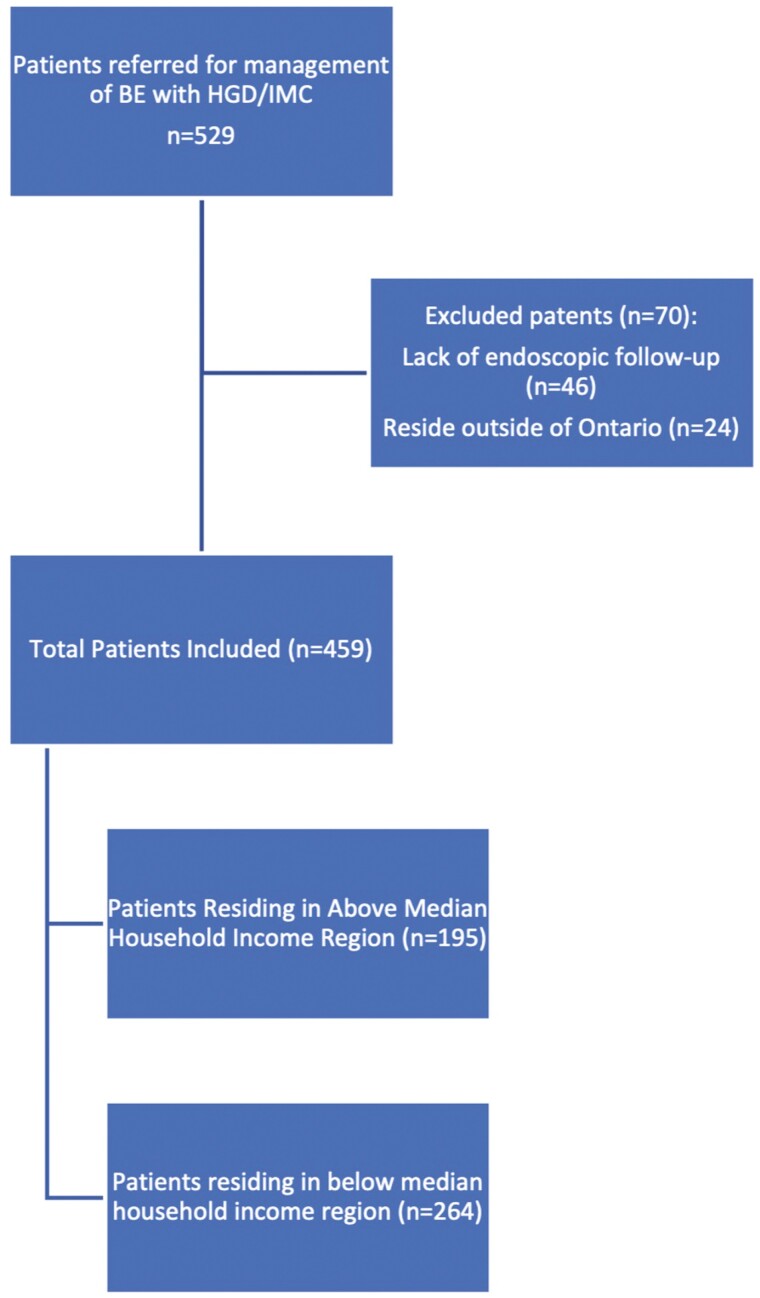
Patient inclusion.

A total of 529 consecutive patients referred for BE with HGD/IMC were reviewed for inclusion. Twenty-four patients were excluded as they resided outside the province of Ontario. Forty-six patients were excluded because of a lack of follow-up endoscopy data before 2 years of follow-up (Figure 1). In total, 459 patients were included for analysis in the study (Table 1).

**Table 1.** Patient demographics and outcomes comparing patients residing in below and above median provincial household income census districts

Characteristics	Overall cohort (*n* = 459)	Above median provincial household income census region (*n* = 195)	Below median provincial household income census region (*n* = 264)	*P*-value
Mean Age (SD)	65 (10)	66 (11)	66 (11)	0.89
Male Gender	83% (*n* = 381/459)	83% (*n* = 162/195)	83% (*n* = 219/264)	0.97
Caucasian	98% (*n* = 414/424)	98% (*n* = 177/181)	98% (*n* = 237/243)	0.86
Median total after-tax household income (IQR), $CAD	64,110 (23,551)	79,679 (15,001)	56,128 (6,428)	<0.001*
Mean driving distance from institution (SD), km	194 (340)	119 (170)	271 (371)	<0.001*
Mean euclidean distance from institution (SD), km	144 (231)	97 (127)	205 (254)	<0.001*
History of smoking	73% (*n* = 316/431)	70% (*n* = 129/185)	76% (*n* = 187/246)	0.14
Active alcohol use >1 drink per day	28% (*n* = 112/397)	26% (*n* = 43/168)	30% (*n* = 69/229)	0.32
GERD symptoms	41% (*n* = 173/426)	40% (*n* = 74/184)	41% (*n* = 99/242)	0.89
Median BMI (IQR), kg/m^2^	28 (6.6)	29 (5.0)	28 (7.7)	0.03*
Hypertension	54% (*n* = 233/431)	55% (*n* = 101/184)	53% (*n* = 132/247)	0.77
Dyslipidemia	54% (*n* = 234/432)	58% (*n* = 108/185)	51% (*n* = 126/247)	0.13
Diabetes	24% (*n* = 103/431)	28% (*n* = 51/185)	21% (*n* = 52/246)	0.12
Use of Aspirin	31% (*n* = 135/433)	33% (*n* = 61/185)	30% (*n* = 74/248)	0.49
Use of Statins	47% (*n* = 204/434)	53% (*n* = 98/186)	43% (*n* = 106/248)	0.04*
Presence of Hiatus Hernia	86% (*n* = 392/455)	87% (*n* = 169/194)	85% (*n* = 223/261)	0.61
Median maximal length of BE (IQR), cm	3.0 (4.0)	3.0 (4.0)	3.0 (4.3)	0.83
Long segment BE	68% (*n* = 310/459)	68% (*n* = 132/195)	67% (*n* = 178/264)	0.95
Median time to treatment (IQR), days	63 (65)	57 (53)	73 (67)	0.42
Median number of EET ­sessions (IQR)	3.0 (3)	4.0 (4)	3.0 (2)	0.20
Achieved CE-IM at 1 year	23% (*n* = 106/456)	23% (*n* = 44/195)	24% (*n* = 62/261)	0.77
Achieved CE-IM at 2 years	35% (*n* = 161/456)	35% (*n* = 69/195)	35% (*n* = 92/261)	0.98
Achieved CE-D at 1 year	46% (*n* = 209/459)	48% (*n* = 94/195)	44% (*n* = 115/264)	0.32
Achieved CE-D at 2 years	59% (*n* = 271/459)	65% (*n* = 127/195)	55% (*n* = 144/264)	0.02*
Invasive esophageal adenocarcinoma	15% (*n* = 70/459)	11% (*n* = 22/195)	18% (*n* = 48/264)	0.04*
Median time to CE-IM (IQR), days	400 (701)	351 (730)	430 (696)	0.25
Median time to CE-D (IQR), days	179 (327)	176 (346)	191 (318)	0.54

^*^Denotes statistical significance, *p*-value <0.05

The overall cohort’s mean age was 65 (SD 10), for which 83% (*n* = 381/459) were male. Median total after-tax household income was $64110 (IQR 23551) of the entire cohort.

Median BMI was 28 kg/m^2^ (IQR 6.6) and 86% (*n* = 392/455) had a hiatus hernia seen on endoscopy. Median maximal BE length was 3.0 cm (IQR 4.0), with 68% (*n* = 310/459) having long-segment BE. Comparing patients from above and below median household income districts, both cohorts were a similar age, gender, race, smoking and alcohol use, length of BE, and the presence of comorbidities. Significant differences were seen in mean driving distance from our institution (119 km versus 271 km in above and below median income districts respectively, *P* = <0.001), BMI (29 kg/m^2^ versus 28 kg/m^2^ in above and below median income districts respectively, *P* = 0.03), and the use of statins (53% versus 43% in above and below median income districts respective, *P* = 0.04). Endoscopic treatment parameters, including time to treatment and median number of EET were similar between groups.

The median time from referral to treatment for the entire cohort was 63 days (IQR 65). 35% (*n* = 161/456) of the cohort achieved CE-IM at 2 years, 59% (*n* = 271/459) achieved CE-D at 2 years, and 15% (*n* = 70/459) were found to have invasive non-endoscopically curable EAC. The median time to CE-IM was 400 days (IQR 701) and the median time to CE-D was 179 days (IQR 327).

### Treatment Outcomes Based on Income Divisions

Rates of CE-IM at 1 year (23% versus 24%, *P* = 0.77) and 2 years (35% versus 35%, *P* = 0.98) were similar between both income cohorts (Table 1). However, the rate of CE-D at 2 years was significantly different between groups, with 65% of patients residing in above-median income census districts achieving CE-D versus 55% of those residing in below-median income census districts, *P* = 0.02. Most notably, the rate of development of invasive non-endoscopically curable EAC was significantly higher (18%, *n* = 48/264) in those residing in below-median income census districts than those residing in above-median income census districts (11%, *n* = 22/195), *P* = 0.04. Of those reaching CE-IM and CE-D, median time to achieve this was similar between groups.

### Factors Associated with CE-IM and CE-D

On univariate analysis, older age (OR 0.97; 95% confidence interval [CI] 0.95 to 0.99) and presence of a hiatus hernia (OR 0.43; 95% CI 0.25 to 0.74) were associated with decreased odds of achieving CE-IM (Tables 2 and 3). The presence of a longer segment of BE (OR 0.23; 95% CI 0.15 to 0.35) was associated with failure to achieve CE-IM. On multivariate analysis, only older age (OR 0.97; 95% CI 0.95 to 0.99) and presence of a longer segment of BE (OR 0.21; 95% CI 0.13 to 0.34) were associated with failure to achieve CE-IM. Residing in below-median household income districts was not associated with CE-IM (OR 0.99; 95% CI 0.64 to 1.55).

**Table 2.** Regression analysis for factors associated with CE-IM at 2 years

	Univariate analysis		Multi-variate analysis	
Factor	Unadjusted OR (95% CI)	*P*-value	Adjusted OR (95% CI)	*P*-value
Age at referral	0.97 (0.95–0.99)	0.001*	0.97 (0.95–0.99)	0.001*
Male Gender	0.75 (0.45–1.23)	0.25	1.14 (0.63–2.06)	0.68
Driving distance from Institution	1.00 (1.00–1.001)	0.34	-	-
BMI	1.03 (1.00–1.06)	0.07	1.03 (0.99–1.07)	0.13
Smoking	0.76 (0.49–1.19)	0.23	0.91 (0.55–1.52)	0.72
Non-Caucasian	0.78 (0.20–3.05)	0.72	0.77 (0.16–3.64)	0.74
Active alcohol use >1 drink per day	0.89 (0.56–1.42)	0.62	-	-
Hypertension	0.94 (0.63–1.39)	0.75	-	-
Dyslipidemia	0.78 (0.52–1.12)	0.22	-	-
Diabetes	0.81 (0.50–1.29)	0.37	-	-
Hiatus Hernia	0.43 (0.25–0.74)	0.002*	0.65 (0.34–1.25)	0.19
Use of Aspirin	0.95 (0.62–1.46)	0.82	-	-
Use of Statins	0.75 (0.50–1.12)	0.16	-	-
Long segment BE	0.23 (0.15–0.35)	<0.001*	0.21 (0.13–0.34)	<0.001*
Time to initial treatment	1.00 (0.99–1.00)	0.16	-	-
Residing in below median income district	0.99 (0.67–1.47)	0.98	0.99 (0.64–1.55)	0.98

^*^Denotes statistical significance, p-value <0.05.

**Table 3.** Regression analysis for factors associated ith CE-D at 2 years

	Univariate Analysis		Multi-variate Analysis	
Factor	Unadjusted OR (95% CI)	*P*-value	Adjusted OR (95% CI)	*P*-value
Age at referral	0.98 (0.96–1.00)	0.03*	0.98 (0.96–1.00)	0.09
Male Gender	0.83 (0.50–1.37)	0.46	1.20 (0.67–2.13)	0.54
Driving Distance from Institution	1.00 (0.99–1.00)	0.94	-	-
BMI	1.04 (1.01–1.08)	0.03*	1.04 (1.00–1.08)	0.05
Smoking	0.66 (0.42–1.04)	0.07	0.71 (0.43–1.16)	0.17
Non-Caucasian	0.66 (0.19–2.30)	0.51	0.66 (0.17–2.55)	0.55
Active Alcohol Use >1 Drink Per Day	0.68 (0.43–1.05)	0.08	-	-
Hypertension	1.10 (0.75–1.62)	0.62	-	-
Dyslipidemia	0.83 (0.57–1.22)	0.35	-	-
Diabetes	0.87 (0.56–1.36)	0.54	-	-
Hiatus Hernia	0.81 (0.47–1.40)	0.45	1.40 (0.71–2.74)	0.33
Use of Aspirin	0.97 (0.64–1.46)	0.87	-	-
Use of Statins	0.83 (0.57–1.22)	0.35	-	-
Long segment BE	0.37 (0.24–0.56)	<0.001*	0.26 (0.15–0.45)	<0.001*
Time to initial treatment	1.00 (0.99–1.00)	0.24	-	-
Residing in below median income district	0.64 (0.44–0.94)	0.02*	0.64 (0.42–0.97)	0.04*

^*^Denotes statistical significance, p-value <0.05.

On univariate analysis, older age at referral (OR 0.98; 95% CI 0.96 to 1.00), presence of long-segment BE (OR 0.37; 95% CI 0.24 to 0.56), and residing in a below-median household income district (OR 0.64; 95% CI 0.44 to 0.94) had a decreased odds of achieving CE-D. Higher BMI (OR 1.04; 95% CI 1.01 to 1.08) was associated with achievement of CE-D. On multivariate analysis, only presence of long segment BE (OR 0.26; 95% CI 0.15 to 0.45) and residing in a below median household income district (OR 0.64; 95% CI 0.42 to 0.97) had decreased odds of achieving CE-D. On sensitivity analysis (Supplementary Table 2) with division of household income into quartiles, only the second lowest income quartile (income $55,147 to $62,089) appeared to be associated with failure to achieve CE-D (OR 0.51; 95% CI 0.28 to 0.92).

### Predictors of Invasive EAC

On univariate analysis, the presence of long-segment BE had higher odds of invasive EAC in our cohort (OR 2.13; 95% CI 1.14 to 3.96) (Table 4). Additionally, those residing in a below-median household income district had higher odds of discovering invasive EAC (OR 1.75; 95% CI 1.02 to 3.01). On multivariate analysis, both factors remained the only significant predictors of invasive EAC. On sensitivity analysis (Supplementary Table 3) with division of household income into quartiles, the second lowest income quartile (income $55,147 to $62,089) approached significance for association with the development of invasive EAC (OR 2.21; 95% CI 1.00 to 4.90).

**Table 4.** Regression analysis for factors associated with non-endoscopically curable invasive esophageal adenocarcinoma

	Univariate analysis		Multi-variate analysis	
Factor	Unadjusted OR (95% CI)	*P*-value	Adjusted OR (95% CI)	*P*-value
Age at referral	1.00 (0.98–1.03)	0.82	1.00 (0.97–1.02)	0.81
Male Gender	1.46 (0.69–3.08)	0.32	1.13 (0.50–2.59)	0.77
Driving Distance from Institution	1.00 (0.99–1.01)	0.51	-	-
BMI	0.98 (0.93–1.02)	0.31	0.98 (0.93–1.03)	0.36
Smoking	1.73 (0.89–3.37)	0.11	1.58 (0.78–3.23)	0.21
Non-Caucasian	0.64 (0.08–5.17)	0.68	0.68 (0.08–5.63)	0.72
Active Alcohol Use >1 Drink Per Day	0.76 (0.40–1.45)	0.41	-	-
Hypertension	0.80 (0.47–1.35)	0.40	-	-
Dyslipidemia	1.66 (0.96–2.88)	0.07	-	-
Diabetes	1.07 (0.58–1.98)	0.82	-	-
Hiatus Hernia	1.24 (0.56–2.74)	0.59	1.03 (0.40–2.68)	0.95
Use of Aspirin	1.25 (0.72–2.18)	0.43	-	-
Use of Statins	1.43 (0.84–2.42)	0.18	-	-
Long segment BE	2.13 (1.14–3.96)	0.02*	2.19 (1.05–4.56)	0.04*
Time to initial treatment	1.00 (0.99–1.00)	0.55	-	-
Residing in below median income district	1.75 (1.02–3.01)	0.04*	1.84 (1.01–3.35)	0.04*

^*^Denotes statistical significance, p-value <0.05.

## DISCUSSION

Our study has found that residing in lower household income districts is independently associated with worse outcomes in patients referred with advanced Barrett’s associated dysplasia. Despite similar baseline medical profiles, patients from lower household income districts had a lower likelihood of achieving CE-D at 2 years and were more likely to be found to have invasive EAC after referral to our institution. As our study was conducted in a universal healthcare system, our results suggest systemic inequities in the institutional care pathways of patients with advanced BE that requires further investigation and quality improvement/policy measures to address.

Higher SES has traditionally been regarded as a risk factor for developing BE (15). However, its impact on individuals with established BE is less clear. A few prior studies evaluating the risk of progression of non-dysplastic BE have suggested an increased risk of progression with lower SES and social class (16–18). Amongst patients with advanced BE who are at the highest risk of progression to invasive EAC and require intensive ETT and follow-up, it is conceivable that the barriers presented by low SES status will have significant impacts on outcomes, as shown by our results. These barriers to access are likely multifactorial at the patient, practitioner, and institutional levels, even in a universal healthcare system where payments for medical care are not an issue.

Prior studies have found that patients with BE are provided with little information and have a poor understanding of its implications (19–21). Anagnostopoulos et al. had shown that 22% of patients diagnosed with BE were given no information on the condition, and some were unaware they had BE (20). This barrier in communication and comprehension is amplified in patients from low SES backgrounds who disproportionately have poorer health literacy (22,23). Amongst patients with esophageal cancer, communication and comprehension difficulties were identified as a significant factor leading to disparities in cancer outcomes in low SES groups (22). Cassani et al. had found that in patients with BE referred for EET, those who were seen in clinical consultation first, rather than direct open-access endoscopy, were more likely to adhere to therapy (24). Thus, adequate time for discussion should be ensured, particularly with patients from low SES groups. Potential strategies include routine pre-endoscopy consultations, dedicated BE clinics/services and utilizing patient facilitators (22,24,25). The growth of virtual care and telehealth may help address disparities related to remoteness or transportation access (26). However, care should be taken not to overlook those who may lack access to reliable phone or computer services (27).

Lower SES status is more prevalent in rural areas (6). Amongst our patient cohort, 68% (n=132/264) of patients residing in above-median household districts versus only 33% (n=86/195) in low-median household districts lived within 100 km of our referral center. The lack of local specialized services and distances to referral institutions present major challenges to accessing care for patients in rural communities that are magnified by low SES status (28). Tinmouth et al. have previously found a significant regional variation in the incidence of EAC in the province of Ontario, where our study is based. Those living outside of the greater Toronto area (GTA) had up to a sixfold increased risk of developing EAC compared to those in the central area (29). Our group also demonstrated that residing within the GTA was associated with a shorter time to receiving ETT for advanced BE (56 days vs. 78 days, p=0.006) (30). Although the cost of physician services is generally not a barrier for Canadians, travel costs can be a significant limitation to accessing specialized care (31). Across Canada, publicly funded medical travel subsidy programs exist but vary widely in what is covered (32). Applying for these programs can be complex and best navigated with social worker assistance (33). Many patients are unaware of these financial assistance programs when they are initially diagnosed with conditions that may incur high out-of-pocket costs (32). Additionally, some patients with below median income may not qualify for social assistance and subsidy programs due to their income being higher than program thresholds. This possibility is suggested by the results of our sensitivity analysis, where the poorest outcomes were seen in the second lowest household income quartile rather than the lowest. Although many studies have shown improved outcomes for BE when patients are treated at expert centers, these specialized services are usually centralized in urban centers (34,35). Thus, care pathways for patients with dysplastic BE should consider and incorporate access to financial/social services for those in low SES and/or rural communities to reduce potential barriers to care.

Adherence to established quality indicators in the management of BE has been a topic of interest in recent years. Several studies demonstrate poor adherence to diagnosis, surveillance, and treatment recommendations (5,36). Amongst patients from low SES backgrounds, there may be increased disparities in adherence to quality standards. Many conventional quality improvement measures fail to consider or address the impacts of SES factors in proposed institutional plans and models (37). At the provider-patient level, perceptions regarding low SES status can significantly influence clinical decision-making, resulting in delayed testing, decreased specialist referrals, and less aggressive therapy (38,39). For patients with advanced BE, the intensive surveillance and treatment protocols present unique challenges for those with low SES backgrounds. Limited sick leaves and uncertainty regarding employment security can significantly impact adherence (22). Although no studies have evaluated the impact of low SES on adherence to quality indicators and treatment in BE, Lineback et al. found that in patients with EAC, those from low SES backgrounds were offered surgical treatment at a significantly lower rate (22). In Ontario, direct associations between SES and EAC survival had been noted that were not present after adjusting for covariates, suggesting care inequities and patient-level confounders as being major factors (40). As practitioners and institutions push toward providing quality care, the recognition of SES disparities should be present in quality improvement proposals where adjustments may need to be made for low SES populations (37).

Our study is the first and largest to evaluate the impacts of low SES in patients with advanced BE. However, several limitations require acknowledgement. Our study design was retrospective and based at a single center, thus limiting its generalizability. However, as our center is the largest BE referral center in the province, our findings are likely applicable to other regions across Canada where, similarly, BE services are centralized at a few provincial centers that service a wide urban and rural catchment area. Additionally, as our study was conducted over a long period, management recommendations and the availability of treatment and surveillance technology may have changed. In particular, the use of high-definition white light endoscopes and virtual chromoendoscopy, as well as stronger evidence to support the use of EET in patients with LGD, may have impacted the timing of neoplasia detection and referral decisions in the community (4). As many of our patients were referred from other locales, we could not assess patient records regarding prior surveillance and management of their BE before being referred to our center.

## CONCLUSION

Low SES status can impact treatment outcomes in patients with advanced BE, with increased risks of developing invasive EAC after referral and failure to achieve CE-D. Numerous factors likely contribute to this inequality, including inadequate communication, misaligned perceptions of care, long distances from referral centers, and financial concerns. Further research is needed to evaluate the root causes and barriers to access for patients with BE from low SES backgrounds to influence policymaking and quality improvement measures.

## Supplementary Material

gwad018_suppl_Supplementary_Material

## Data Availability

The data, analytic methods and study materials underlying this article will be shared on reasonable request directly to the corresponding author.
