# Supplementary material for: Impact of Residing in Below Median Household Income Districts on Outcomes in Patients with Advanced Barrett’s Esophagus
Source: J Can Assoc Gastroenterol. 2023 Apr 27;6(4):137–44. doi: 10.1093/jcag/gwad018 (PMC10395662; doi:10.1093/jcag/gwad018)
Supplement: gwad018_suppl_Supplementary_Material [file gwad018_suppl_supplementary_material.docx]

**Supplementary Table 1.** Multivariate Regression Analysis for Factors Associated With CE-IM at 2 years with Household Median Income Divided via Quartiles

|  | **Multi-variate Analysis** | |
| --- | --- | --- |
| **Factor** | **Adjusted OR (95% CI)** | **P-value** |
| Age at referral | 0.97 (0.95-0.99) | 0.002* |
| Male Gender | 1.07 (0.59-1.95) | 0.83 |
| BMI | 1.03 (0.99-1.07) | 0.11 |
| Smoking | 0.93 (0.55-1.54) | 0.77 |
| Non-Caucasian | 0.64 (0.14-3.05) | 0.58 |
| Hiatus Hernia | 0.64 (0.33-1.24) | 0.19 |
| Long segment BE | 0.21 (0.13-0.34) | <0.001* |
| Household Median Income Quartile | | |
| 1^st^ Quartile (<$55,147) | 0.75 (0.40-1.42) | 0.38 |
| 2^nd^ Quartile ($55,147 – $62,089) | 0.66 (0.36 – 1.22) | 0.19 |
| 3^rd^ Quartile ($62,090 – $75,056) | 0.43 (0.23 – 0.82) | 0.01* |
| 4^th^ Quartile ($75,057 -$119,672) | 1 | - |

**Supplementary Table 2.** Multivariate Regression Analysis for Factors Associated With CE-D at 2 years with Household Median Income Divided via Quartiles

|  | **Multi-variate Analysis** | |
| --- | --- | --- |
| **Factor** | **Adjusted OR (95% CI)** | **P-value** |
| Age at referral | 0.98 (0.96-1.004) | 0.11 |
| Male Gender | 1.18 (0.66-2.12) | 0.57 |
| BMI | 1.04 (1.00-1.08) | 0.03* |
| Smoking | 0.70 (0.43-1.16) | 0.16 |
| Non-Caucasian | 0.68 (0.18-2.61) | 0.57 |
| Hiatus Hernia | 1.44 (0.73-2.83) | 0.29 |
| Long segment BE | 0.26 (0.15-0.43) | <0.001* |
| Household Median Income Quartile | | |
| 1^st^ Quartile (<$55,147) | 0.68 (0.37-1.26) | 0.22 |
| 2^nd^ Quartile ($55,147 – $62,089) | 0.51 (0.28 – 0.92) | 0.03* |
| 3^rd^ Quartile ($62,090 – $75,056) | 0.96 (0.52 – 1.76) | 0.88 |
| 4^th^ Quartile ($75,057 -$119,672) | 1 | - |

**Supplementary Table 3:** Multivariate Regression Analysis for Factors Associated with Non-endoscopically Curable Invasive Esophageal Adenocarcinoma with Household Median Income Divided via Quartiles

|  | **Multi-variate Analysis** | |
| --- | --- | --- |
| **Factor** | **Adjusted OR (95% CI)** | **P-value** |
| Age at referral | 1.00 (0.97-1.02) | 0.71 |
| Male Gender | 1.17 (0.51-2.69) | 0.71 |
| BMI | 0.97 (0.92-1.02) | 0.25 |
| Smoking | 1.59 (0.78-3.26) | 0.21 |
| Non-Caucasian | 0.63 (0.08-5.32) | 0.67 |
| Hiatus Hernia | 1.00 (0.39-2.60) | 0.99 |
| Long segment BE | 2.29 (1.10-4.78) | 0.03* |
| Household Median Income Quartile | | |
| 1^st^ Quartile (<$55,147) | 1.27 (0.54-2.99) | 0.59 |
| 2^nd^ Quartile ($55,147 – $62,089) | 2.21 (1.00 – 4.90) | 0.050 |
| 3^rd^ Quartile ($62,090 – $75,056) | 0.88 (0.36 – 2.16) | 0.78 |
| 4^th^ Quartile ($75,057 -$119,672) | 1 | - |
